# Dr. Robert Ogden Doremus

**Published:** 1906-04

**Authors:** 


DR. ROBERT OGDEN DOREMUS, of New York, died at his home Mach 22, 1906, after an illness of ten days, at the age of eighty years. He graduated in medicine at the New York University in 1850, having received the degree of A.B., from the same institution in 1842. He was one of the earliest teachers of chemistry to establish laboratory instruction in this country. Dr. Doremus may be said to have possessed the genius of instruction in his chosen field, and, withal, was an inventor and discoverer along the lines of his special branch of science.

It would be impossible in the narrow space to which this notice is limited to do justice to the career of this many-sided, talented man; but the following editorial comment from *The New York Tribune* is a worthy tribute in a few well-chosen words that may well be rewritten as an expression of our respect to his memory.

"It is doubtful if any other man ever served the metropolis so usefully and so honorably for so long a time as the late Robert Ogden Doremus. More than sixty years intervened between the beginning and close of his career as an educator, though his connection with the City College and the medical school of Bellevue Hospital covered a period of only two-thirds that length. His field of usefulness was by no means limited to instructing college students in chemistry and toxicology. He was famous for his original researches, for devising modern methods of analysis where the criminal use of poison was suspected and for public lectures and experiments which went far to popularize science. Lovers of music, too, were under great obligations to him. His endeavors to secure for New York the presentation of works which would gratify his own passion and critical taste conduced directly and indirectly to the delight of thousands of others."

